# Learning to heal minds in the eastern Mediterranean

**DOI:** 10.2471/BLT.16.021016

**Published:** 2016-10-01

## Abstract

Training health workers and other professionals to provide mental health care builds local capacity to respond to these needs in the eastern Mediterranean. Dale Gavlak reports.

“There are so many people in need of care,” says Dr Dyaa Saymah, who until recently served as the World Health Organization’s (WHO) mental health officer for Gaza. “Depression, anxiety and post-traumatic stress disorder are common in both Gaza and the West Bank.”

“The massive destruction and losses in 2014 increased the number of those already wounded physically and psychologically and in need of care and support,” Saymah says, referring to one of three conflicts in the last decade that brought death and destruction to Gaza.

Humanitarian emergencies and conflict in many parts of WHO’s Eastern Mediterranean Region, stretching from Morocco to Pakistan, is increasing the need for mental health care.

It is mainly because of such emergencies, in more than half of its 22 countries and territories, that this WHO Region has the highest prevalence of mental disorders, specifically depressive illness and anxiety disorders, according to the *Global Burden of Disease* study.

“During and after complex emergencies, the rates of mental disorders increase significantly and a large proportion of the population experiences psychological and emotional distress,” says Dr Khalid Saeed, Regional Adviser for mental health and neurological disorders at WHO’s Regional Office for the Eastern Mediterranean in Cairo. 

“At these times there is a particularly great need for mental health and psychosocial support interventions to be integrated into the health and social sector response programmes,” he says.

“Countries in this region have made progress towards integrating … mental health into primary care.”

Khalid Saeed

“Countries in this region have made progress towards integrating such mental health into primary care,” Saeed says. The idea is to make mental health care available through primary care providers to reach people in need with the appropriate support and care.

“The extent of this progress varies between countries, irrespective of country income, but still an estimated 76–85% of people in need are not receiving the care and treatment they need,” he says.

Several countries in the region have started to extend mental health care to more people by training physicians, nurses and other health professionals who do not specialize in mental health care by using WHO’s mental health Gap Action Programme Intervention Guide (mhGAP–IG).

The mhGAP–IG explains how nurses can provide clinical care for depression, psychosis, bipolar disorders, dementia and other common mental health conditions. So far Afghanistan, Egypt, Iraq, Jordan, Kuwait, Lebanon, Pakistan, Qatar, Somalia, the Syrian Arab Republic, Tunisia, and the West Bank and Gaza are using the manual.

In the West Bank and Gaza a comprehensive mental health care reform was introduced in 2003 with WHO technical support and European Union funding.

New stand-alone community mental health care centres have been established – 13 in the West Bank and six in Gaza – and the number of people who visit these is increasing every year, according to health ministry annual reports. In addition, 40 out of 54 government primary care centres in Gaza now provide mental health care.

The reform is bringing a shift from centralized psychiatric hospitals to a community-based model, recommended by WHO, that integrates mental health care into primary care. The latest addition to the mental health care landscape in the West Bank is a two-year master’s degree programme in psychotherapy, one of the few of its kind in the Eastern Mediterranean region.

The programme opened its doors in January to the first group of 15 students, all of whom are already working in the field of mental health, to train them formally in psychological interventions such as cognitive behavioural therapy. The theoretical part of the course is based at Al Quds University and it includes more than 500 clinical hours of practice supervised by tutors.

The new master’s degree programme is the brainchild of Palestinian community activist Fadwa Abbad, who has been training social workers in health clinics and community centres in the Bethlehem area for the last 20 years.

Abbad felt that there was a large but unmet need for mental health services of high quality in the West Bank. She conducted a needs assessment that confirmed her fears and showed that not all psychotherapists and counsellors there met international standards.

“Palestinians are living in a situation of constant stress, but many people are not getting the support they need to cope,” Abbad says. “I worked with friends and colleagues in the fields of psychotherapy and clinical psychology to create the master’s degree programme to address these gaps.”

Abbad set up Sunna al-Amal, a nongovernmental organization (NGO) in 2014 in the West Bank, to train Palestinian psychotherapists. The NGO joined forces with al-Quds University to provide the first master’s degree study programme in psychotherapy for students from the West Bank.

Abbad hopes that the master’s degree programme will attract students from Gaza next year too.

“Palestinians have experienced a great deal of adversity and there aren’t enough qualified people to provide psychotherapy and behavioural therapy,” says Malika (not her real name), a student on the course who enrolled in January after completing a bachelor’s degree in social work in the United States of America.

As in the rest of the Arab world, one of the challenges for providing mental health care is that seeking psychological help in Gaza and the West Bank carries a social stigma.

For Dr Fahmy Hanna, a technical officer from the Department of Mental Health and Substance Abuse at WHO headquarters in Geneva, another of the challenges is having enough qualified people to provide mental health care – something the course helps to address.

“Nearly one in 10 people worldwide suffer from a mental disorder and, globally, there are only seven psychologists working in mental health for every one million people, Hanna says. “But the ratio in the Eastern Mediterranean region is significantly lower. There are only four psychologists working in mental health per one million people.”

In addition to training general health-care professionals, other specialists such as community workers, social workers and teachers can also provide basic versions of cognitive-behavioural therapy, interpersonal therapy and other types of mental health support, Hanna says. “We have evidence that this approach works and we would like to scale this up in low-income settings.

“This approach, combined with training general health workers to use the WHO mhGAP–IG to treat common and severe mental disorders, means we can reach substantially more people in need,” Hanna says.

WHO is also providing support to other parts of the region that are facing conflict and emergencies.

In Libya, WHO worked with national and international mental health experts to adapt the mhGAP–IG training programme to the local context and needs in their country.

“The needs are higher in adverse situations, so when you introduce a new service, you need to work with the communities not only to let them know that a new service exists but also to combat the misconceptions about mental disorders,” Hanna says.

Raising community awareness of mental health needs and combating stigma have proved vital in both Libya and the Syrian Arab Republic.

Gaza and the West Bank, already have a community-based mental health infrastructure. “By training young and talented professionals who are placed in various community centres and who join the Ministry of Health workforce, the whole system changed,” Saymah says.

“Today, if you go to a community health centre, the doctor can refer people to psychologists for cognitive behavioural therapy sessions and you can find psychotherapy sessions offered by psychologists. 

“Many people coming to these mental health clinics are not on medication, but coming for psychotherapy and benefiting from this a lot,” Saymah says.

“A cornerstone for developing good mental health care is training good mental health practitioners.”

Dyaa Saymah

“A cornerstone for developing good mental health care is training good mental health practitioners,” Saymah says.

“That’s why we invested so much in developing those people. I believe they will be the leaders of the future mental health reform in Gaza.”

**Figure:**
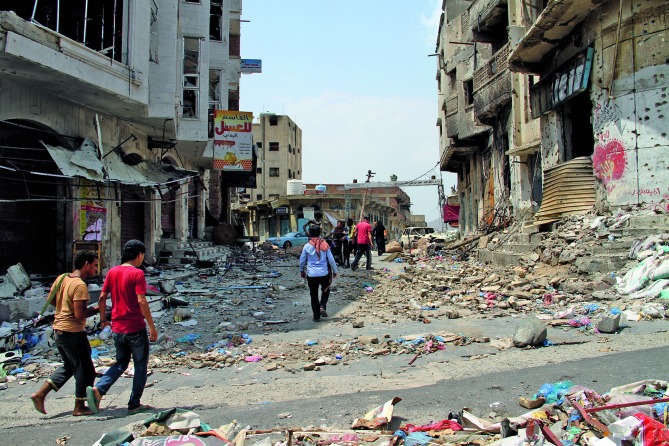
Destruction of buildings during the fighting in Taiz, Yemen in 2015. Yemen is one of several countries in the WHO Eastern Mediterranean Region experiencing conflict  and humanitarian emergencies.

ICRC/Wael Al-Absi

